# Next generation secondary electron detector with energy analysis capability for SEM

**DOI:** 10.1111/jmi.12867

**Published:** 2020-02-17

**Authors:** A. SURI, A. PRATT, S. TEAR, C. WALKER, M. EL‐GOMATI

**Affiliations:** ^1^ Department of Electronic Engineering University of York York U.K.; ^2^ Department of Physics University of York York U.K.; ^3^ York Probe Sources Ltd Harwood road York U.K.

**Keywords:** Auger electron spectroscopy, Bessel Box, electron detector, electron microscopy, energy analyser, energy filtered images, scanning electron microscope

## Abstract

We report the working of a novel detector design based on a Bessel Box (BB) electron energy analyser in a scanning electron microscope (SEM). We demonstrate the application of our detector for elemental identification through Auger electron detection in an SEM environment and its potential as a complementary technique to energy dispersive X‐ray (EDX) spectroscopy. We also demonstrate energy‐filtered secondary electron imaging of a copper‐on‐silicon sample using an electron pass energy of 12 eV.

**Lay Description:**

Advancements in the field of the Scanning Electron Microscopy have been one of the major nanotechnology enablers. A Scanning Electron Microscope (SEM) generates a magnified image of the sample by bombarding it with an electron beam and detecting the electrons that scatter off the surface along with the electrons that are generated in the sample. Conventional detectors such as the Everhart‐Thornley detector (ET) or through‐the‐lens (TTL) detectors, either offer little to no energy analysis (ET) or limited energy filtering capability (e.g the low‐pass energy filter in TTL). This information is crucial to interpret the image of the sample under study. What is needed is a smart and compact detector that can detect electrons and furnish energy inside the SEM chamber. Here, we report a novel secondary electron (SE) detector design with energy analysis capability for use in scanning electron microscopes. The detector is based on the design of a Bessel Box (BB) energy analyser. We have designed and experimentally tested it in an SEM environment. The band‐pass filter action of the detector enables the BB to be operated at a selected energy and allows a narrow window of energies to be detected for generating energy‐filtered images.

## Introduction

Auger electron spectroscopy (AES) is a widely established technique for elemental identification on solid surfaces (Prutton & El Gomati, 2006). However, it is normally carried out in an ultra‐high vacuum (UHV) environment to maintain a clean sample surface for the duration of the experiment (El‐Gomati *et al*., 2011). Conventionally, energy dispersive X‐ray spectroscopy (EDX) is performed for elemental identification in a scanning electron microscope (SEM) but not AES. However, EDX has inferior spatial resolution and depth of information (∼1 µm) when compared to AES where it is better than 10 nm (Goldstein, 2003). Furthermore, recent developments in energy‐filtered secondary electron microscopy (Masters *et al*., 2015; Masters *et al*., 2019) (EFSEM) has laid emphasis on the low energy secondary electron (SE) spectra. Conventional SEM detectors such as the Everhart–Thornley detector (ETD) or through‐the‐lens (TTL) detectors, both offer little to no energy analysis (e.g. ETD (Everhart & Thornley, 1960)) or limited energy filtering capability [e.g. the low‐pass energy filter in TTL (Kazemian *et al*., 2007), limited energy‐range (SE spectrum) band‐pass capability as in Konvalina *et al*. (2019); Kursheed (2010)]. Here, we focus on electron energy analysis in an SEM for spectroscopy (SE and AES) and microscopy (SE) applications. We report a novel detector design based on the Bessel Box (BB) electron energy analyser.

## The Bessel Box electron energy analyser

**Figure 1:**
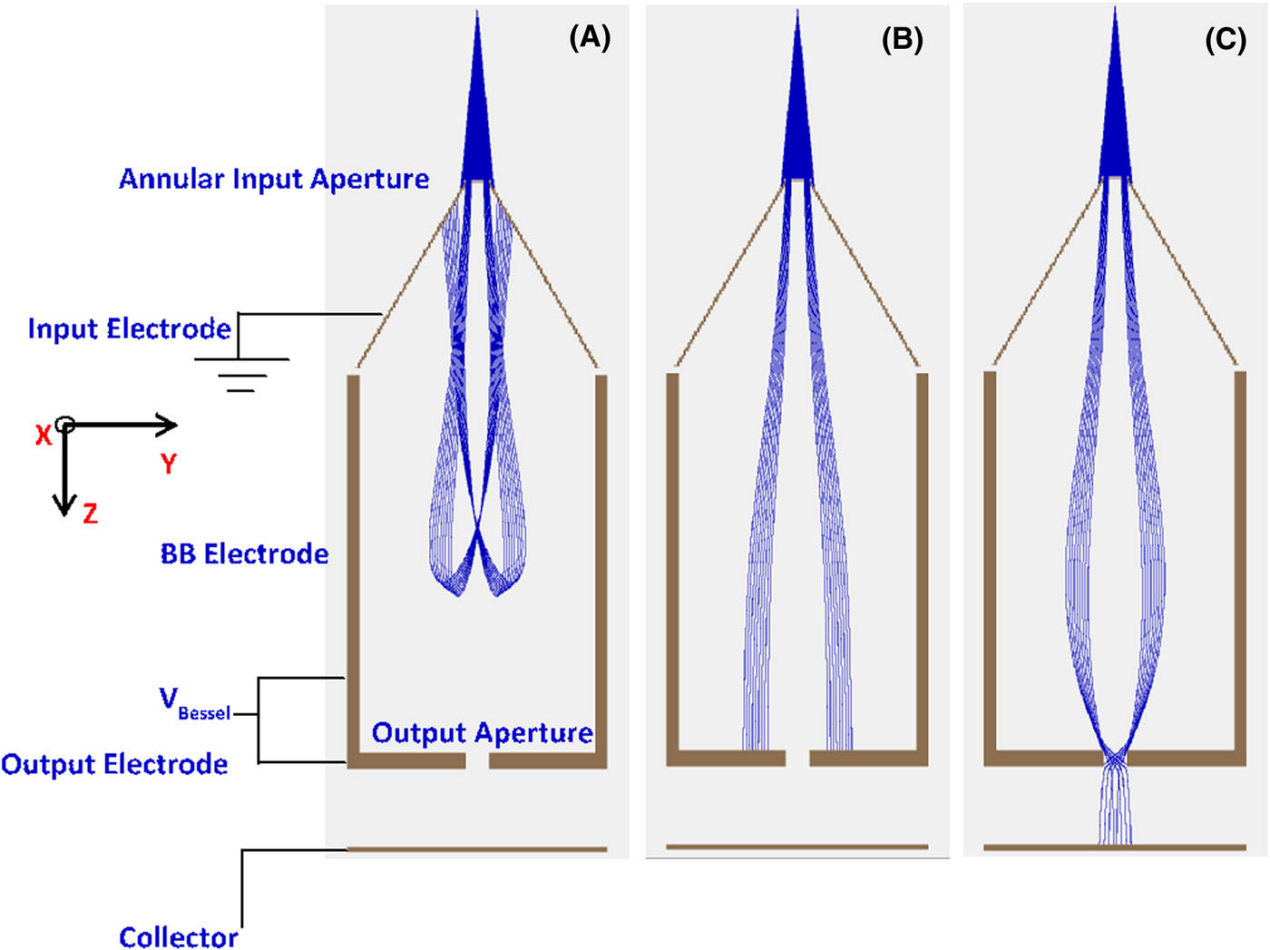
Simulations done in SIMION 8.1 (Dahl, 2000) of electron trajectories in the BB. (A) Lower energy electrons have insufficient energy to reach the collector. (B) The BB is not able to focus higher energy electrons. The on‐axis trajectories are blocked using an annular input aperture. (C) For a given set of voltages the BB focuses a narrow energy range of electrons at the output aperture before they are collected.

The BB is so‐called because the variations in the potentials and fields inside the BB depend on the mathematical modified‐Bessel function (Allen *et al*., 1976). The BB has a simple cylindrical geometry, comprising of three electrodes: central, input and output electrodes. The apertures are defined at the input/output electrodes. An annular input aperture is used to block the on‐axis trajectories from transmitting through the BB. This is needed because the on‐axis trajectories are not affected by the internal fields of the BB. The input electrode is grounded whilst the central and output electrodes are shorted together and negatively biased for the operation of the BB in retarding configuration, as proposed by Schiwietz *et al*. (2015). We build upon this design by making the BB very compact and modifying the flat geometry of the input electrode into a conical shape (Fig. 1). This is to take the geometrical constraints of the sample chamber in an SEM into account. The BB operates in a band‐pass configuration as depicted in Figure 1. Electrons with energies lower than the peak pass energy are repelled back by the retarding fields (Fig. 1A), higher electron energies are terminated at the walls (Fig. 1B) and only a narrow range of electron energies are allowed to transmit through for collection (Fig. 1C). We have already reported the simulation of electron trajectories and subsequent experimental verification in a test chamber elsewhere (Suri *et al*., 2019).

The present prototype design has a length of 50 mm and a diameter of 40 mm. The energy resolution of the BB analyser has been numerically calculated and experimentally verified to be 0.4 % with a collection efficiency of 0.3%. This design approaches the energy resolution of a cylindrical mirror analyser (CMA) of 0.3%, but with a reduced collection efficiency due to lack of 100% azimuth angular collection. Higher collection efficiencies are reported for analysers such as the CMA (16%) and toroidal analyser (20%) (Kursheed, 2010). The BB design has a large depth of focus of 12 mm which ensures the local topography variations in the sample remain in focus with the BB, making it an attractive candidate as a detector for an SEM. We also report the field of view of the device to be 400 µm × 400 µm. The simplicity of construction and the compactness of design enables the BB detector to be used as an ‘add‐on device’ over SEM variants. This has been demonstrated in this report through experimental spectra carried out in a UHV test chamber and JEOL 7000F SEM.

## Results and discussion

**Figure 2:**
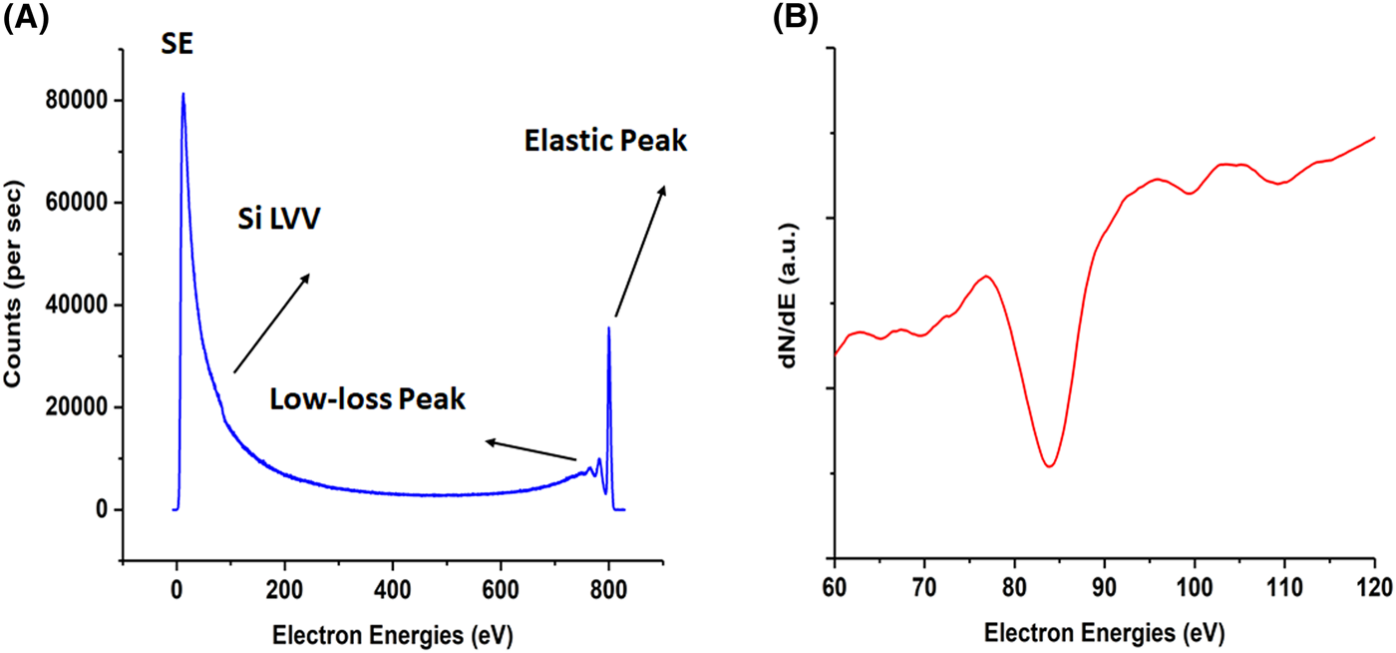
(A) Electron spectrum acquired from the bombardment of an 800 eV electron beam on a clean silicon (111) sample. (B) The differentiated silicon Auger peak.

Preliminary testing of the BB was carried out in a UHV system furnished by a thermionic electron gun. The UHV chamber was maintained at a base pressure of 10^−8^ Pa. The BB detector was mounted on an xyz sample manipulator with a geometry in which the detector and the electron beam were orthogonal to each other and the sample was tilted at 45° with respect to the BB and the electron beam axis. An n‐doped Si(111) sample was cleaned by flash annealing to a maximum temperature of 1200°C. A multimodal spectrum with low energy SE peak at 12 eV and elastic peak corresponding to the primary beam energy at 800 eV with a beam current of the order of 50 nA was then acquired, as shown in Figure 2(A). Primary beam electrons that have undergone low‐energy losses, such as plasmons, can also be seen near the elastic peak. On the high energy side of the SE peak, a shoulder is observed which corresponds to the Si LVV Auger peak. Figure 2(B) shows the Si LVV peak at 84 eV in the differentiated spectrum.

**Figure 3:**
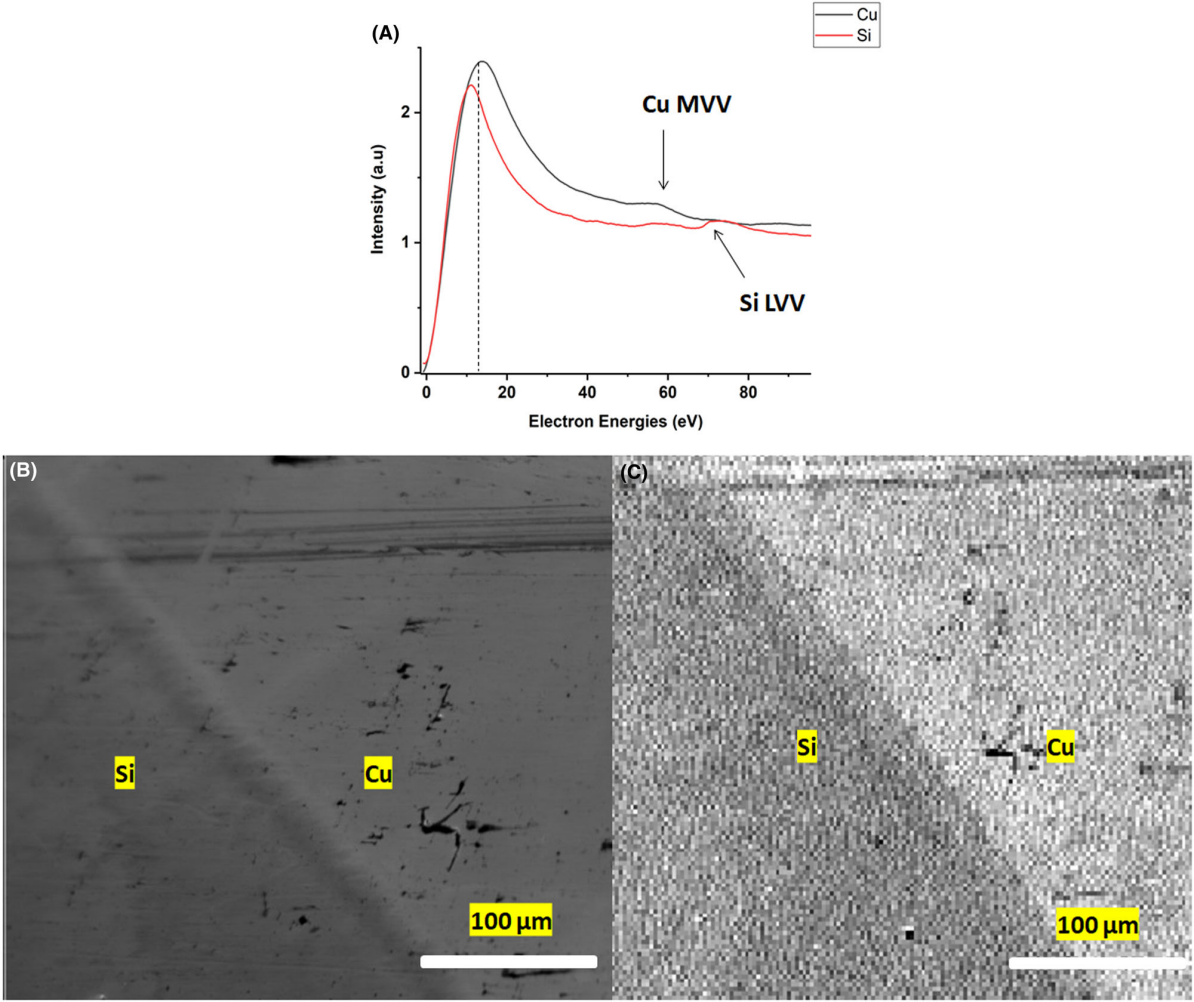
(A) The SE spectra from a copper‐on‐silicon sample, as shown in the ETD image in (B). The features observed on the low energy spectra are Si and Cu Auger peaks. (C) The energy‐filtered image obtained with the BB detector tuned to a 12 eV pass energy.

Following the preliminary experiments in the UHV system, the BB was mounted on a JEOL 7000F field‐emission SEM. The BB‐sample geometry was kept similar to the UHV experiment: the sample was mounted at 45° with respect to the BB detector and the electron gun axes. The sample consisted of a 100 nm thick layer of copper deposited on a silicon substrate. The sample was plasma cleaned *ex situ* before inserting into the SEM. Plasma cleaner from Diener Electronic GmbH was used to clean the sample. The sample was cleaned at 25% instrument power for 3 min and then inserted into the SEM. Figure 3(B) shows a SE image, obtained using the microscope's ETD, of the copper‐on‐silicon sample. Firstly, narrow energy spectra (0–100 eV) were acquired using the BB detector from the Si and Cu regions. The electron beam energy used was 10 keV with a beam current of the order of 15 nA. The low energy spectra acquired from both regions are normalised to the SE peak intensity as shown in Figure 3(A). The small peaks on the SE background seen are low energy Si and Cu Auger peaks at 80 eV and 57 eV, respectively. For SE filtered imaging, the BB detector was tuned to a pass energy of 12 eV. Figure 3(C) shows a 256 × 256 pixel resolution image of the same area (400 µm × 400 µm) as acquired for the ETD image of Figure 3(B). As for the ETD image, in the BB image the Cu region is brighter in contrast than the Si region. Generally, this is related to the relative differences in the secondary electron yields (SEY) of copper and silicon (Walker *et al*., 2008). However, by acquiring the energy‐filtered BB image at 12 eV, with the additional AES capability, it could potentially be used to explain the origin of the contrast mechanism.

**Figure 4:**
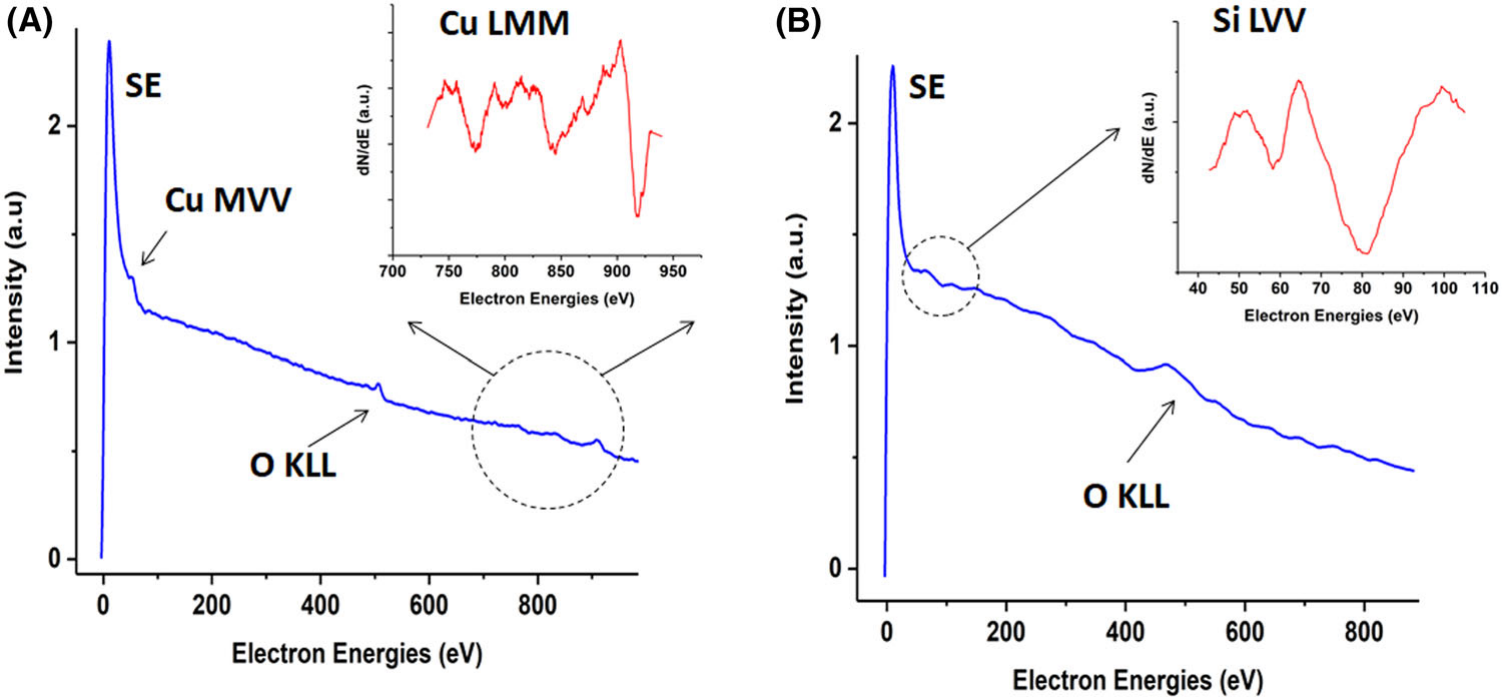
Auger electron spectroscopy in an SEM environment. (A) Electron spectra acquired from a copper region with the inset showing higher energy Cu Auger electron peaks. (B) The spectrum acquired from the Si region with the inset showing the low energy Si Auger electron peak. Both insets are the differentiated part of the spectra.

A wider energy scan was acquired from the Si and Cu regions by operating the BB in the spectroscopic mode. Cu and O Auger electron peaks were detected from the Cu region as seen in Figure 4(A). The inset shows the differentiated high‐energy Cu Auger peaks. Similarly, Si and O Auger electron peaks were observed from the Si region. The quantification of an Auger peak intensity in an SEM environment is likely to be difficult because of the thin impurity/adsorbate layer normally present in such an environment. Nonetheless, the energy position of the Auger electron peaks acquired using the BB detector has offered opportunities to employ AES detection in an SEM environment as a complementary technique to EDX for elemental identification with some potential for higher spatial resolution.

## Conclusion

We have designed and experimentally tested a novel SE detector that is also able to analyse electron energies. The band‐pass filter action of the detector enables the device to be operated at a selected energy and allows a narrow window of energies to be detected for energy‐filtered SE microscopy. Furthermore, we have also demonstrated the acquisition of Auger electron peaks in an SEM environment, a technique complementary to EDX in an SEM for elemental identification, with some potential for higher spatial resolution. The results obtained here show that with current technology of plasma cleaning, AES data could be collected in an SEM within a high vacuum sample environment. This should be useful in many applications. The compact design of this BB allows it to be mounted as an add‐on device across SEM variants.
